# Epidemiology and clinical outcomes of microbial keratitis in South East Nepal: a mixed-methods study

**DOI:** 10.1136/bmjophth-2022-001031

**Published:** 2022-06-20

**Authors:** Lila Raj Puri, Helen Burn, Abhishek Roshan, Ramanand Biswakarma, Matthew Burton

**Affiliations:** 1 The Fred Hollows Foundation, Alexandria, New South Wales, Australia; 2 London School of Hygiene and Tropical Medicine Faculty of Infectious and Tropical Diseases, London, UK; 3 Sagarmatha Choudhary Eye Hospital, Lahan, Nepal; 4 National Institute for Health Research Biomedical Research Centre for Ophthalmology, Moorfields Eye Hospital NHS Foundation Trust, London, UK

**Keywords:** Cornea, Infection, Epidemiology, Public health, Vision

## Abstract

**Objective:**

To describe the epidemiology of microbial keratitis in patients presenting to a tertiary eye hospital in South East Nepal alongside qualitative interviews exploring patient perspectives on barriers to accessing eye care services.

**Methods and analysis:**

All patients with microbial keratitis (>16 years) presenting to Sagarmatha Choudhary Eye Hospital, Nepal between 1 May 2017 and 31 July 2017 were recruited. Data were collected on patient demographics, precipitating factors and pathway to care. Clinical examination was performed and microbiological samples collected. Visual acuity was measured at final follow-up. Semistructured interviews and focus group discussions explored the patient journey and barriers to accessing care.

**Results:**

We recruited 174 participants; 88 (51%) were male (mean age of 47 years) and 126 (72%) were farmers. Ocular trauma with vegetative matter was reported by 79 (45%) and 84 (48%) had fungal infections. Visual acuity was <3/60 in 107 (61%) of affected eyes at presentation, reducing to 73 (42%) at last follow-up. Factors associated with poor visual outcome were trauma with vegetative matter, delayed presentation and poor visual acuity at presentation. Qualitative interviews with 40 patients identified lack of awareness of the disease and available services, poor knowledge and practice of community health workers and lack of affordability and accessibility of treatment as important barriers.

**Conclusion:**

The epidemiology of microbial keratitis in this region was similar to other tropical regions. Patient interviews highlighted need for public health awareness campaigns on microbial keratitis, training of community health staff on the urgency of this condition and improvements in accessibility and affordability of ocular treatments.

**What is already known on this topic?**

- Microbial keratitis is a leading cause of blindness and ocular morbidity globally. Low-income and middle-income countries are disproportionately affected often due to tropical climates, higher incidence of ocular trauma and inadequate access to treatment.

**What this study adds?**

- The epidemiology data add to sparse global literature on microbial keratitis by describing the incidence, microbiology and treatment of microbial keratitis in the Terai region of Nepal. This is the first study to accompany the epidemiological data with qualitative data describing patient and healthcare provider barriers to providing timely and appropriate treatment for microbial keratitis.

**How this study might affect research, practice or policy?**

- By understanding patient and healthcare provider barriers interventions can be focused on key parts of the patient journey to improve visual acuity outcomes from this serious ocular disease. In particular, this study highlights the need for training for community healthcare workers in the referral and initial management of this condition, improving patient awareness and availability of affordable and effective treatments.

## Introduction

In 2017, 1.3 million people were estimated to be bilaterally blind from corneal opacity (excluding trachoma and vitamin A deficiency), accounting for 3.2% of global blindness.^1^ Globally, it is thought to cause approximately 2 million cases of monocular blindness per year and is the second leading cause of blindness affecting one eye in tropical regions after cataract.^2^ Microbial keratitis (MK) has been described as a ‘silent epidemic’ and is a major public health problem in low-income and middle-income countries (LMICs) where patients often suffer prolonged morbidity, loss of vision, pain and stigma.^3^ Nepal has one of the highest incidences globally for fungal keratitis estimated to be 73 per 100 000.^4^

MK is defined as loss of the corneal epithelium, with underlying stromal infiltration and suppuration associated with signs of inflammation with or without hypopyon. MK is an ocular emergency that requires prompt diagnosis and appropriate management to ensure the best visual outcome for the patient. Without adequate treatment, corneal infection leads to blindness through corneal scarring, corneal perforation and loss of the eye. LMICs are disproportionately affected by MK, largely because of ocular trauma from contaminated objects such as vegetative matter, poor access to appropriate eye care and persistence of communicable diseases such as trachoma, onchocerciasis and leprosy.

Sagarmatha Choudhary Eye Hospital (SCEH), situated in south east Nepal, is a high-volume tertiary eye hospital providing eye care service to the people of eastern region of Nepal and northern India. The hospital examines around 200 000 patients and performs more than 50 000 surgeries annually. MK is the most common condition seen in the cornea outpatient department.

To date, most studies on MK in Nepal have primarily evaluated epidemiological features, predisposing factors and clinical features of corneal ulceration. There are no qualitative studies to identify the factors responsible for poor visual outcome and causes for delayed presentation to hospital for treatment. The aim of this study is to describe the epidemiology of MK, determine factors associated with a poor outcome and suggest approaches to improve patient outcomes in this area.

## Materials and methods

A mixed-methods quantitative and qualitative study, including focus group discussions (FGDs) and semistructured interviews (SSIs), was carried out in the SCEH cornea department between 1 May 2017 and 31 July 2017.

### Quantitative study design

The quantitative component of the study was conducted to describe the epidemiology and clinical outcomes of MK. MK was defined as a corneal epithelial defect with an underlying corneal stromal infiltrate, and signs of acute inflammation. All Nepali patients 16 years and older attending the SCEH corneal department between 1 May 2017 and 31 July 2017 with a diagnosis of MK were invited to take part. We excluded patients not willing to participate, those under age 16 years and patients attending from India. Patients with presumed viral or protozoal keratitis (based on history and clinical examination) were also excluded. A standardised form was completed for each patient documenting socio-demographic information, clinical findings including duration of symptoms, prior treatment, time between onset of symptoms and presentation to SCEH, previous ophthalmic history and treatment received at SCEH.

Presenting visual acuity was measured using a Snellen chart. Visual Acuity was categorised using WHO classification of presenting visual acuity.^5^ An ophthalmologist (LRP) examined the eyes using a slit lamp. The size of the ulcer was measured as the maximum dimension of the ulcer after fluorescein staining. The size of the stromal infiltrate and depth of ulcer were also recorded. The presence and height of hypopyon was measured.

Corneal scraping was performed using a sterile Bard-Parker blade (No. 15). The procedure was performed at the slit lamp or with binocular loupes, following instillation of preservative-free 2% lignocaine hydrochloride. Material obtained from scraping of the leading edge and base of each ulcer was smeared onto two slides, one stained with Gram stain and the other with 10% potassium hydroxide for direct microscopic evaluation.

The patients were treated as per the hospital treatment for corneal infections, after clinical and laboratory diagnosis. Cases of keratitis caused by Gram-positive bacteria were treated with a combination of fortified cefazolin and fortified gentamicin eye-drops and Gram-negative bacteria with ciprofloxacin 0.3% eye-drops. Fungal keratitis was treated with natamycin 5% eye-drops. Patients were counselled regarding the use of medications and followed up for next 48 hours. The frequency of the medications was adjusted depending on the clinical response.

The patients were followed up at 3 weeks postdischarge from hospital. Visual acuity was measured, and size of ulcer, size and depth of stromal infiltration and presence of hypopyon were recorded. The visual acuity outcomes were subdivided into: (1) ‘good outcome’ of VA 6/60 or better at final visit; (2) ‘poor outcome’ if VA <6/60 at final visit.

### Qualitative study design

The qualitative component of the study was conducted to evaluate health seeking behaviour of MK patients and explore the barriers to accessing services. A non-probabilistic, purposive sampling strategy was used. The ophthalmologist working in the cornea clinic recruited patients to be interviewed either as SSIs or part of an FGD. Patients continued to be recruited until no new views arose during the interviews and saturation was reached.

Participants could use their preferred language which was either Nepali, Hindi or Maithili. All interviews were recorded using a voice recorder and afterwards transcribed and translated into English. All interviews had a facilitator and a translator present. Twenty in-depth SSIs were carried out each lasting approximately 15–20 min. FGDs (two male and two female) were conducted in a meeting room within the premises of the SCEH using a topic guide.

### Statistical analysis

Data were entered into MS Excel 2016. Cleaning of the data was done and STATA V.14 (StataCorp) was used for analysis. Descriptive statistics showing numbers and percentages were used to display sociodemographic and clinical characteristics of participants. Univariate logistic regression analysis was used to determine the clinical and sociodemographic correlates of a poor visual outcome following attendance at the hospital with MK. A 95% CI was used as a measure of precision for the estimated prevalence ratios. A process of familiarisation and reflection was carried out to identify key themes in the data. Following this, coding was done; derived by group, gender and page of the transcript. Quotes were identified to support the themes and subthemes.

## Results

### Participants

We recruited a total of 174 Nepali patients presenting with MK to the SCEH Cornea Department between 1 May 2017 and 31 July 2017. Sociodemographic characteristics are shown in table 1. The mean age was 47 years (SD 14.7), there were nearly equal proportions of males (51%) and females (49%). The most frequent occupation was farming (72%) followed by labour worker (13%). The majority of patients (80%) came from the Terai (plain) region. The most common predisposing factor was ocular trauma, which was reported by 103 (59%) patients, of whom 79 (77%) had sustained an injury involving vegetative material (table 2). More than half of the ulcers had evidence of fungal infections (n=84, 48%), or mixed fungal and bacterial infections (n=31, 18%), with a minority having evidence of bacteria only infections (n=39, 22%) (table 2).

**Table 1:** Sociodemographic characteristics of participants

Characteristics	Frequency (%)
Age (years)	
Mean (SD)	47 (14.7)
16–30	35 (20)
31–40	28 (16)
41–50	34 (20)
51–60	48 (27)
61–70	28 (16)
71–80	1 (1)
Gender	
Male	88 (51)
Female	86 (49)
Occupation	
Farmer	126 (72)
Labour work	23 (13)
Housewife	15 (9)
Student	7 (4)
Office work	3 (2)
Geographic location	
Terai	140 (80)
Hills	27 (16)
Mountains	7 (4)

**Table 2:** Presentation and clinical characteristics of participants

Characteristic	Frequency (%)
Ocular trauma	
Trauma	103 (59)
Vegetative matter	79 (77)
Non-veg matter	24 (23)
No trauma	71 (41)
Duration of symptoms prior to attending SCEH	
Median days (IQR)	15 (6–30)
<5 days	19 (11)
5–14 days	59 (34)
15–30 days	83 (48)
>30 days	13 (7)
Reasons for delayed presentation	
Distance	94 (54)
Money	28 (16)
Nobody to accompany	24 (14)
No information about eye hospital	15 (9)
No response	13 (7)
Consultation before hospital visit	
Ophthalmic assistant/optometrist	83 (48)
Local pharmacy	74 (43)
Traditional Healer	8 (5)
Ophthalmologist	7 (4)
Self	2 (1)
Treatments used before hospital visit*	
Antibiotic eye-drops	135 (78)
Antifungal eye-drops	66 (38)
Corticosteroid eye-drops	37 (21)
Traditional eye medicine	15 (9)
No Medication	2 (1)
Visual acuity at presentation	
6/5–6/12	18 (10)
6/18	9 (5)
6/24–3/60	40 (23)
<3/60	107 (61)
Visual acuity at final follow-up	
6/5–6/12	22 (13)
6/18	7 (4)
6/24–3/60	72 (41)
<3/60	73 (42)
Types of organism	
Fungus	84 (48)
Bacteria	39 (22)
Mixed	31 (18)
No organism found	20 (12)
Size of ulcer	
<2 mm	43 (26)
2–5 mm	104 (62)
>5 mm	21 (13)
Missing data	6
Stromal depth	
<20%	41 (24)
20%–50%	117 (70)
>50%	10 (6)
Missing data	6

*Some participants were using more than one type of medication at presentation.

SCEH, Sagarmatha Chaudhary Eye Hospital.

Forty patients were recruited to the qualitative component of the study; 20 took part in SSIs and 20 in FGDs. The mean age of participants in the SSIs was 47 (SD 14.2), 8 (40%) were female and 12 (60%) were male, 7 (35%) were early presenters (<15 days) and 13 (65%) were late presenters (>15 days). Four FGDs were carried out with five participants in each group. Two FGDs were all male with mean age 48, one comprising of late presenters (>15 days) and one early presenters (<15 days). Similarly, two FGDs were all female with mean age 43. These FGDs were also divided into a late presenter group (n=5) and an early presenter group (n=5).

### Delayed presentation

The median number of days between onset of symptoms and presenting at the SCEH cornea department was 15 days (IQR 6–30). A large majority of patients (n=155, 89%) presented five or more days after symptom onset. The primary reported reasons for delayed presentation are shown in table 2. These were further supported by the reported explanation in the qualitative work.

Lack of awareness about the disease:

I never thought in my wildest dream that such a small event (dust particle into the eye) could cause such dangerous problem to my eyes. (CUP-5)

Unavailability of local eye care service:

There is no eye care service available near my village. I have to travel the full day to reach the nearest centre. (CUP-4)

Cost of services and treatment:

I’m late because of money problem. I am the only earning member in the family. I had to sell my goat to arrange money for the treatment. (CUP-20)

Late referral from place of their initial consultation:

I went to the medical shop in my village one day after I started having pain and redness in right eye. He gave me some eye drop and I used it for five days. But there was no improvement. He gave another drop. But still no improvement. Only after ten days did he tell me that I should go to hospital for better treatment. (FGD/Male/Late presenter)

Lack of knowledge about available eye services:

I had no idea where to go for the treatment. First, I went to traditional healer and then to local medical store. After two weeks one of my neighbour told me to go to eye hospital for treatment. (CUP-12)

Lack of family support/nobody to accompany:

I am very old and I can’t work now. My children are busy in their work and they don’t take enough care of me. I told them about my problem after four days when I could not tolerate the pain, but they ignored it. Finally, when my vision was gone and my eye looked white, they brought me here after 12 days. (CUP-8)

Faith in traditional healers:

I have this problem in the eye because of a witch. So, I went to jhakri (traditional healer) for treatment without telling my husband. He treated me with herbs and mustard oil [into the eye] for five days. But I was losing vision day by day and there was whitish appearance in my eye. Then my husband brought me here for treatment. (FGD/Female /late presenter)

### Consultations prior to presenting to hospital eye clinic

We asked participants whether they had sought help in other locations before presenting to SCEH (table 2); the most common primary consultations were with an ophthalmic assistant or optometrist (n=83, 48%), or at a local pharmacy (n=74, 43%). Only 8 (5%) people reported having been to see a traditional healer about their eye problem. The majority of patients were using antibiotic eye-drops prior to attending SCEH (n=135, 78%); and over half were using either antifungal eye-drops or corticosteroid eye-drops (table 2).

After seven days, we went to Mirchaiya eye clinic. We got eye medicines used for 3 days and again went there, but not improved then we came to Lahan. (CUP-9)

First I got an eye drop from medical store. It was not improving, then I went to eye doctor after 20 days (CUP-16)

We believe in God, so we feel that the traditional healer can only get rid of the curse from our body. So, we always go to them first. (CUP-9)

### Clinical outcomes

Over half of patients presenting to SCEH with MK had a presenting visual acuity of <3/60 in the affected eye (n=107, 61%). This was reduced to 73 patients (42%) with <3/60 at their last visit (table 2). A poor visual acuity outcome (defined as <6/60) at final follow-up was found to be associated with trauma with vegetative matter (OR 2.28 95% CI 0.90 to 5.76, p=0.08), delayed presentation to the hospital (OR 3.37 95% CI 1.19 to 9.50, p=0.02) and poor visual acuity on presentation (OR 2.69 95% CI 0.97 to 7.44, p=0.05) (table 3).

**Table 3:** Factors associated with a poor visual acuity outcome in the affected eye (presenting visual acuity <6/60 vs ≥6/60)

Variables	Visual acuity outcome		OR	95% CI	P value
	Good (≥6/60) n (%)	Poor (<6/60) n (%)			
Ocular trauma					
Non-vegetative matter trauma	13 (54.2)	11 (45.8)	1	–	–
Vegetative matter trauma	27 (34.2)	52 (65.8)	2.28	(0.90 to 5.76)	0.08
Duration of symptoms					
Median duration (IQR)	12 (6–20)	15 (9–30)			
<5 days	12 (63.2)	7 (36.8)	1	–	–
5–14 days	22 (37.3)	37 (62.7)	2.88	0.99 to 8.41	0.05
15–30 days	28 (33.7)	55 (66.3)	3.37	1.19 to 9.50	0.02
>30 days	5 (38.5)	8 (61.5)	2.74	0.64 to 11.75	0.17
Consultation before hospital					
Local pharmacy	31 (41.9)	43 (58.1)	1	–	–
Ophthalmic assistant/optometrist	31 (37.4)	52 (62.7)	1.21	0.64 to 2.29	0.56
Ophthalmologist	2 (28.6)	5 (71.4)	1.80	0.33 to 9.90	0.49
Traditional healer	2 (25.0)	6 (75.0)	2.17	0.41 to 11.4	0.36
Self	1 (50.0)	1 (50.0)	0.72	0.04 to 12.0	0.82
Corticosteroid eye-drops prior to presentation					
None used	51 (76.1)	84 (78.5)	1	–	–
Corticosteroid used	16 (23.9)	23 (21.5)	0.91	0.48 to 1.74	0.78
Gender					
Male	35 (52.2)	53 (49.5)	1		
Female	32 (47.8)	54 (50.5)	1.11	0.60 to 2.05	0.73
Age					
16–30	17 (48.6)	18 (51.4)	1		
31–60	40 (36.4)	70 (63.6)	1.65	0.77 to 3.56	0.20
61–80	10 (34.5)	19 (65.5)	1.79	0.65 to 4.94	0.26
Presenting visual acuity					
6/5–6/12	9 (50.0)	9 (50.0)	1		
6/18	5 (55.6)	4 (44.4)	0.80	0.16 to 3.99	0.79
6/24–3/60	24 (60.0)	16 (40.0)	0.67	0.22 to 2.04	0.48
<3/60	29 (27.1)	78 (72.9)	2.69	0.97 to 7.44	0.05

### Improving outcomes and reducing barriers

We asked participants about approaches that they thought might lead to improving outcomes for this problem and reduce barriers to accessing care.

#### Eye health awareness campaign in the community

Patients stated that prior to their diagnosis with MK and visiting the hospital they had limited knowledge about the disease and its sight threatening complications. Many also mention the importance of preventing ocular trauma through eye protection during farm work.

In my village people still believe in the traditional healer for treatment. The hospital should educate them about the disease, its treatment and eye health in general, so that they seek proper advice for their eye problem. (FGD/Male/Early presenter)

People should be educated about eye health conditions. The people should pay attention during their work like harvesting, grinding and should use protective glasses during performing work. (FGD/female/early presenter)

#### Improve the accessibility of eye care service

Most of the patients stated they would have visited the eye hospital earlier if it was nearby. They emphasised the need for local eye care centres to reduce the cost of attending, improve follow-up, improve outcomes and promote early presentation.

There is no eye care service at my place. I would have visited the eye centre early, and hopefully I would have better result, if it was near. (CUP-15)

#### Eye health training for pharmacists, primary healthcare workers and traditional healers

IDIs and FGDs revealed that many patients came late to the hospital because of delayed referral by a pharmacist, medical practitioner or traditional healer. Many identified the need to train these community health workers.

Now I realized that we should not have wasted time in seeking treatment from local medical shop or traditional healer because they are not the right person to treat this type of serious eye problem. (CUP-2)

#### Improve the availability and affordability of medicine

More than half of the patients stated that medicines prescribed in the hospital were not available locally. Many expressed the need for affordable and easily available medicines for better compliance and outcome.

If the medicines are cheap and easily available, I will put the medicine regularly as told by doctors. (CUP-16)

Medicine is not available in our place, only few types of eye drops are available in local medical store. (FGD/male/late presenter)

## Discussion

This study describes the clinical presentation, microbiological diagnoses and visual outcomes of MK in the Terai region of Nepal, alongside patient interviews highlighting the barriers faced in accessing timely and appropriate clinical care. Patients affected by MK in this region tend to be of working age and engaged in agricultural work. This is a common trend among other studies of MK in LMICs including Uganda,^6^ Nepal^7^ and India.^8 9^ Two-thirds of patients were blind in the affected eye on presentation, and almost a half remained blind on their last outpatient visit. Poor visual outcomes from MK in LMICs are common; in Uganda 30% of patients had monocular blindness (visual acuity <3/60) at discharge,^6^ and in Tanzania 66% of patients had VA <6/60 at discharge.^10^

The majority of patients had seen another healthcare professional before attending the tertiary hospital (mostly ophthalmic assistant/optometrist or local pharmacy) and most were taking antibiotic eye-drops on arrival. Previous studies have shown that prompt use of prophylactic ocular antibiotics following a traumatic corneal abrasion can prevent MK from developing and results in much better outcomes.^11–13^ Despite the apparent common use of ocular antibiotics prior to patients presenting at the corneal department, visual outcomes remain poor. This suggests perhaps antibiotics were started too late after MK had already developed rather than being used prophylactically. Treatment for MK needs to be started as early as possible in order to achieve good visual outcomes, and once ulceration is advanced treatment is often ineffective.^14^ Of concern is that 21% of patients were taking corticosteroid eye-drops prior to presentation which can cause worsening of the infective corneal infiltrate, something that has been reported elsewhere.^9^

One predictor of a poor visual outcome at final follow-up was found to be trauma with vegetative matter. Other studies in Nepal and elsewhere in South Asia have also found that MK is commonly preceded by ocular trauma, with fungal keratitis more likely after trauma with vegetative matter leading to worse visual outcomes.^11 15 16^ More than half of all microbiological diagnoses were fungal, in keeping with other studies showing that fungal keratitis accounts for 20%–60% of MK in tropical regions.^17^ Fungal keratitis is challenging to treat and often requires a long and intensive treatment regimen which is difficult to adhere to due to the often limited availability and higher cost of the treatment. Even when promptly and adequately treated, up to 30% of infections still lead to corneal perforations or eye loss.^18 19^Brown *et al* found in their systematic review of literature on fungal keratitis that approximately 100 000 eyes are removed annually from fungal keratitis due to late diagnosis and poor therapeutic outcomes.^20^

The second significant risk factor for a poor visual outcome was delayed presentation from the start of symptoms to the eye hospital, with patients delaying between 15 and 30 days having a three-time increased risk of a poor visual outcome compared with those presenting within the first 5 days (OR 3.37, p=0.02 95% CI 1.19 to 9.50). The reasons for delayed presentation collected in the quantitative data strongly correlated with themes emerging from the qualitative data. Patient interviews provided greater insight into the reasons for delayed presentation to the eye hospital. Four common themes for delayed presentation emerged in the patient interviews which in turn can be used to plan methods to improve the patient journey, clinical experience and improve outcomes.

First, lack of awareness about the seriousness of this condition was common, with patients often not realising the importance of prompt treatment to prevent blindness. Patients highlighted the need for educational events in the community to raise awareness of the importance of accessing prompt treatment for eye trauma and ocular infections and where to find treatment.

Second, patients stated both a lack of available local eye services and a lack of knowledge about what local services were available. Nepal has a well-developed network of community eye care centres that feed into large regional eye hospitals and is fairly unique among LMICs for this organised structure of eye care services. The eye care centres are staffed by ophthalmic assistants, optometrists and eye health workers who have been trained to provide diagnoses, treatment and referrals for common ocular conditions. In Sagarmatha zone, where SCEH is situated, there are seven community eye care centres providing services to approximately 2.06 million people.^21^ As such, much of the population still have a long distance to travel to reach one of these centres, particularly in the hilly regions where there are fewer centres and travel is more challenging. Again, public health campaigns designed by the local eye care centres could help to raise awareness of their services and encourage patients to attend as well as possible long-term plans to increase the number of eye care centres in areas further away from SCEH.

Third, while the majority of patients had a prior consultation in the community before attending SCEH, many stated in their interviews that this consultation had led to delay, incorrect treatment and false reassurance. This finding is supported by a recent study of the knowledge of MK among primary healthcare workers in the same region. We found that only 27% of primary healthcare workers in community health posts could correctly diagnose MK and 59% could correctly treat the condition. Only 69% were aware that it is an ocular emergency requiring prompt treatment and referral.^22^ This study supports the findings from Burn *et al* that primary eye care training is required in the region, particularly focused on common blinding conditions such as MK, in order to avoid delays in referral and harmful medical treatments being offered (such as traditional eye medicines (TEM) and corticosteroid eye-drops).

Lastly, patients described costs, lack of family support and nobody to accompany them as important barriers to accessing eye care. In particular, the medication prescribed to treat fungal keratitis is often expensive and not locally available meaning that patient compliance with treatment can be poor. Improving the availability and affordability of anti-fungal medication, in particular, is a key priority in improving outcomes from MK in this region. A current randomised control trial is investigating the use of a cheap and more widely available medication, chlorhexidine 0.2% eye-drop, as a non-inferior alternative to natamycin 5% eye-drop which is currently first line.^23^ Although on the WHO Essential Medicines List 2017, natamycin 5% eye-drops are often not available in much of sub-Saharan Africa and some Asian countries, and where it is available it is prohibitively expensive.^10^

The main limitation of this study is the potential for selection bias and researcher bias in the qualitative interviews. While we aimed to maintain a neutral position while carrying out the interviews, the personal beliefs and experiences of the interviewer can still influence the response of the participant. As purposive sampling was carried out those participating in the interviews may not be fully representative of participants attending the corneal department at SCEH. Second, corneal samples were not cultured as this facility was not available at the hospital. Culture of corneal scrapes is the gold standard method for identifying bacterial corneal infections. Without this diagnostic tool the microbiological diagnoses have been made only from Gram stain and microscopy limiting the accuracy of the results and could account for a low bacterial detection rate. Lastly, this study only collected data at one tertiary eye hospital in the region and would be improved by expanding the sample to include multiple sites to allow greater generalisability of the results.

## Conclusion

This study is the first to correlate the epidemiology of MK in South East Nepal with the patient perspective on the pathway to accessible treatment. The majority of cases of MK seen in the corneal department were fungal keratitis occurring as a result of ocular trauma in farm workers. Most patients presented late to the eye hospital, with poor visual acuity on arrival reducing their chances of a successful visual outcome. Interviews with patients highlighted several important areas to target in order to improve access to timely and effective medical treatment for this condition; improving patient awareness of the disease and available eye care services, educating the community health workers who provide initial treatment and referral prior to attending the eye hospital, and increasing the affordability and accessibility of available treatment.

## Data Availability

Data are available on reasonable request. Data are available on request.
